# Fourier Motion Processing in the Optic Tectum and Pretectum of the Zebrafish Larva

**DOI:** 10.3389/fncir.2021.814128

**Published:** 2022-01-07

**Authors:** Auriane Duchemin, Martin Privat, Germán Sumbre

**Affiliations:** Institut de Biologie de l'ENS (IBENS), Département de Biologie, Ecole normale supérieure, CNRS, INSERM, Université PSL, Paris, France

**Keywords:** zebrafish, Fourier motion, visual system, two-photon calcium imaging, neuronal circuit dynamics

## Abstract

In the presence of moving visual stimuli, the majority of animals follow the Fourier motion energy (luminance), independently of other stimulus features (edges, contrast, etc.). While the behavioral response to Fourier motion has been studied in the past, how Fourier motion is represented and processed by sensory brain areas remains elusive. Here, we investigated how visual moving stimuli with or without the first Fourier component (square-wave signal or missing fundamental signal) are represented in the main visual regions of the zebrafish brain. First, we monitored the larva's optokinetic response (OKR) induced by square-wave and missing fundamental signals. Then, we used two-photon microscopy and GCaMP6f zebrafish larvae to monitor neuronal circuit dynamics in the optic tectum and the pretectum. We observed that both the optic tectum and the pretectum circuits responded to the square-wave gratings. However, only the pretectum responded specifically to the direction of the missing-fundamental signal. In addition, a group of neurons in the pretectum responded to the direction of the behavioral output (OKR), independently of the type of stimulus presented. Our results suggest that the optic tectum responds to the different features of the stimulus (e.g., contrast, spatial frequency, direction, etc.), but does not respond to the direction of motion if the motion information is not coherent (e.g., the luminance and the edges and contrast in the missing-fundamental signal). On the other hand, the pretectum mainly responds to the motion of the stimulus based on the Fourier energy.

## 1. Introduction

Visual motion signals are composed of several features that the visual system needs to extract to detect movement. The specific features driving motion detection have been extensively studied. Fourier signals, or first-order signals, represent the luminance-defined features of the image, while the non-Fourier (second-order) signals correspond to other features such as edges, contrast, etc. Studies using modified square-wave moving gratings in which the first-Fourier component was suppressed (missing-fundamental signal), showed that the perception of movement is dominated by the Fourier components of the signal. Fourier transform of a pure square-wave results in its fundamental frequency and its odd harmonics (third, fifth, seventh, and so on), such that they have, respectively, amplitudes of one-third, one-fifth, one-seventh, etc. of the amplitude of the fundamental frequency. Using Fourier decomposition, it is possible to create a stimulus that has a Fourier motion energy moving in the opposite direction to that of the other features (edges, contrast, textures, etc.) by removing the fundamental frequency of the square-wave. This stimulus is called the missing-fundamental stimulus (sometimes also depicted as fluted-square-wave) (Adelson and Bergen, 1985; Chen et al., 2005).

In humans, moving the missing-fundamental stimulus induces motion ambiguity. The initial ocular pursuit responses are always in the direction of the Fourier energy, even though other features move in the opposite direction (Chen et al., 2005; Sheliga et al., 2005). The effect is also present in monkeys (Miura et al., 2006) and in mice (Sugita et al., 2012). In zebrafish, the effect is not limited to the initial ocular pursuit responses: when the missing-fundamental stimulus is presented to larvae, they constantly follow the direction of the Fourier energy rather than that of the other features (Orger et al., 2000).

Despite these studies describing the psychophysical effects of Fourier motion and the missing-fundamental signals, their representation in sensory brain areas remains elusive.

Here, we use behavior, two-photon Ca2+ imaging of transgenic zebrafish larvae expressing the genetically encoded Ca2+ indicator GCaMP6f, to monitor the visual responses of the optic tectum and the pretectum to moving grids consisting of square-wave and missing-fundamental signals.

To assess the larva's detection of the direction of the moving stimuli, we used the optokinetic response (OKR). The OKR consists of slow eye rotations (pursuits) in the direction of the detected motion followed by rapid saccades in the opposite direction to reset the eyes position. It occurs in response to whole-field motion and serves to stabilize the external world on the retina of the fish (Huang and Neuhauss, 2008; Portugues and Engert, 2009).

In zebrafish, the optic tectum, homologous to the mammalian superior colliculus, mediates the detection of visual information, integrates multiple sensory modalities (Thompson et al., 2016) and generates goal-directed behaviors such as prey capture (Romano et al., 2015; Förster et al., 2020). The pretectum, homologous to the accessory optic system in mammals (Matsuda et al., 2021), is involved in optic flow processing and controls the optokinetic and optomotor responses (OKR and OMR). It has been shown that the pretectum is necessary and sufficient for the OKR (Kubo et al., 2014). The pretectum integrates monocular information to create binocular representation, that is essential for the optomotor response (Naumann et al., 2016). Wang et al. (2020) showed that the tectum responds mainly to small stimuli in the upper nasal visual field (corresponding to the location of prey during hunting), while the pretectum represents larger stimuli in the lower visual field (optic flow). However, it should be noted that the pretectum has at least two functional regions, one that is responsive to optic flow and another one, more rostral, that is involved in prey detection (Semmelhack et al., 2014). The latter region is retinotopic and innervated by the temporal retina, which creates a high-resolution representation of the anterior visual field (where the preys are located before being captured) (Robles et al., 2014).

Here, we found that the missing-fundamental signal and the square-wave signal, although capable of inducing a similar behavioral output, are processed differently by the larva's visual centers. The optic tectum did not show responses to the direction of the missing fundamental signal, although it did so to the direction of the square-wave signal. In contrast, the pretectum displayed activity specifically associated with the detected direction of motion (optic flow) independently of the type of stimulus presented, including the missing-fundamental signal. Our results suggest that the optic tectum cannot extract the direction of motion from the Fourier energy alone, in case the luminance and the other non-Fourier features of the signal display incoherent or ambiguous directional information. On the other hand, the Fourier energy seems to be sufficient for the pretectum to represent the general direction of optic flow.

## 2. Materials and Methods

### 2.1. Ethics Statement

All experimental procedures were approved by the comité d'éthique en expérimentation animale n°005. Reference number APAFIS#27495-2020100614519712 v14.

### 2.2. Animals

All experiments were performed using zebrafish larvae from 7 to 9 days post-fertilization (dpf), expressing pan-neuronally the GCaMP6f indicator (HuC:H2B-GCaMP6f (from Dunn et al., 2016) on a *nacre* (mitfa -/-) background (Lister et al., 1999). The embryos were collected and raised at 28°C in 0.5x E3 embryo medium (E3 in mM: 5 NaCl, 0.17 KCl, 0.33 CaCl2, 0.33 MgCl2 pH 7.2). Larvae were kept under 14/10 h on/off light cycles and fed after 5 dpf with Paramecia.

### 2.3. Visual Stimulation

The larvae were placed in the center of a chamber surrounded by a screen. Visual stimuli were projected on the screen using a pico-projector (AAXA P4X). The stimulation field covered approximately 180°x60° (azimuth x height) of the larva's visual field. All stimuli were generated using Matlab (The MathWorks, Inc) and the Psychophysics Toolbox Version 3 (Brainard, 1997; Kleiner et al., 2007) extension. A geometrical deformation was imposed on the stimuli to take into account the curvature of the chamber, so not to affect the spatial frequency of the stimulus. To prevent interference of the visual stimulus with the emission of the GCaMP signal, we only used the red light LED of the projector (620 nm), and added to the projector a 561nm long-pass filter (BLP01-561 Semrock). The luminance of the black part of the screen (*Imin*) was 8 lux, and the luminance of the red part of the screen (*Imax*) was 800 lux. The contrast was calculated as 0.98 (Michelson contrast, commonly used for periodic functions: $\frac{\left(Imax-Imin\right)}{\left(Imax+Imin\right)}=\frac{792}{808}=0.98$).

#### 2.3.1. Generation of the Missing-Fundamental and the Square-Wave Visual Stimuli

The square-wave gratings moved with a velocity of 180°/s and each bar corresponded to 16° of visual angle. This stimulus is known to induce the optokinetic response (OKR). To generate the missing-fundamental stimulus, we subtracted the principal Fourier component F1 (fundamental) of the square-wave stimulus (F1 = 0%). When this stimulus is presented in quarter-cycle jumps, it induces in the larva OKR in the opposite direction compared to the physical motion of the edges and features of the stimulus. This is because we create a movement by shifting the frames by quarter-wavelength steps (compared to the original square-wave). In this case, the third harmonic of the signal moves forwards by three-quarters of its own wavelength. In other words, it appears to move backwards by one-quarter of its wavelength. As the third harmonic is also the strongest Fourier component in the missing-fundamental stimulus, the latter appears to the larva as moving backwards as well, even though the rest of its features move forwards. A third stimulus that we projected to the larvae was a square-wave signal with the same velocity but with a spatial frequency of one-third of the first signal (approximately 5.3° of visual angle), that corresponds to the third harmonic of the first signal and to the highest power of harmonic in the missing-fundamental stimulus. This signal also induces OKR in the opposite direction compared to the first square-wave stimulus, but in contrast to the missing-fundamental signal, all its features go in the same direction.

**Figure 1:**
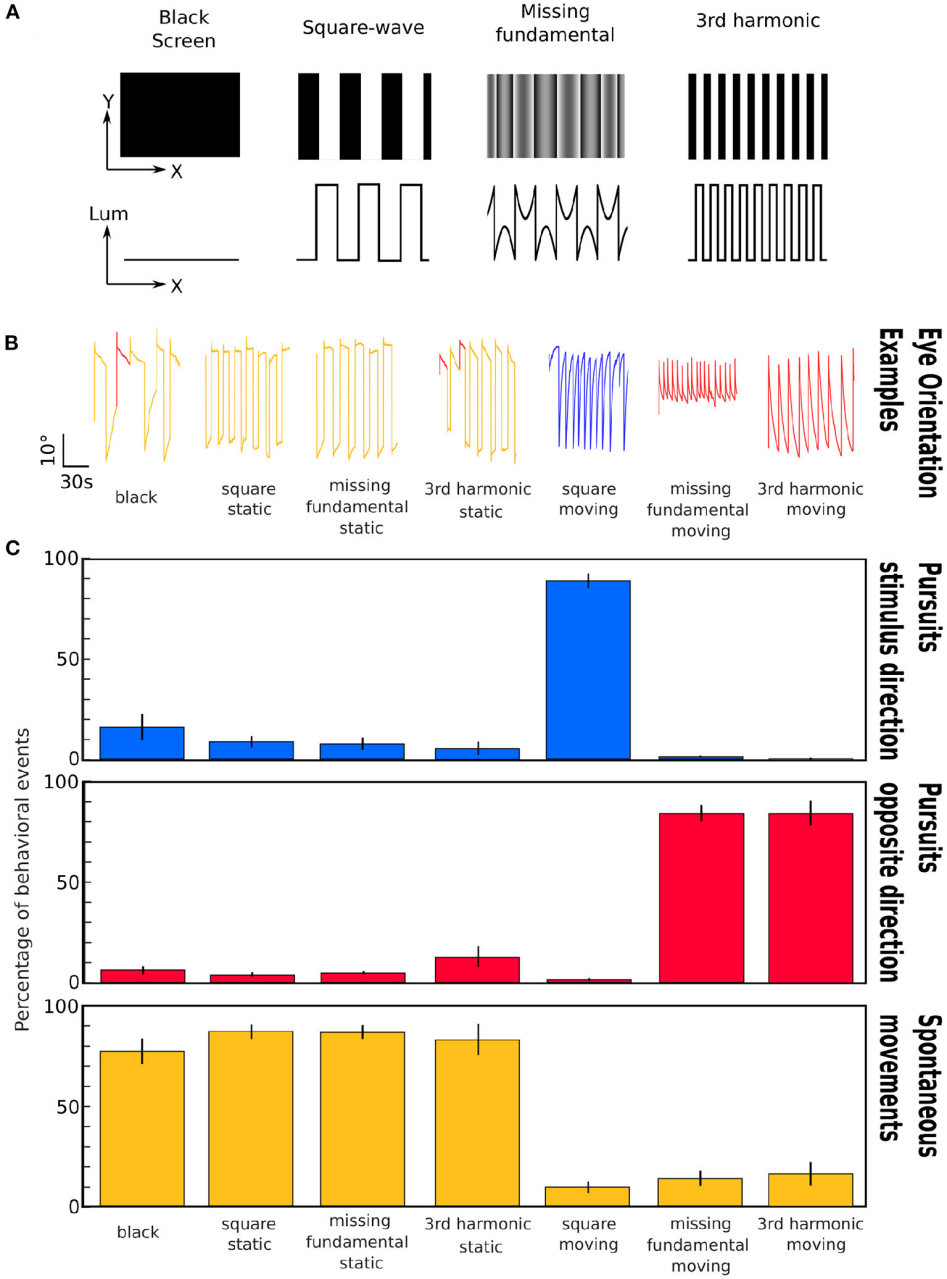
Representation of the different stimuli and induced eye movements. **(A)** Images corresponding to the different stimuli projected to the larvae. First row: visual appearance of the stimuli; Second row: Plot of the luminance as a function of spatial position across the x axis. **(B)** Examples of the eye orientation traces induced by the different types of visual stimuli: black screen, static square-wave signal, static missing-fundamental signal, static 3rd harmonic signal, moving square-wave signal, moving missing-fundamental signal and moving 3rd harmonic signal. Yellow: spontaneous movements; blue: pursuits in the stimulus direction; red: pursuits in the opposite direction. **(C)** Percentage of behavioral events (pursuits in the direction of the stimulus' motion, pursuits in the opposite direction, spontaneous rotations) for the seven presented stimuli. The error bars represent the standard error of the mean. The behavioral experiment was performed on *n* = 6 larvae. Values as means±S.D.: Black: stimulus direction 16.3 ± 15.8%; opposite direction 6.3 ± 5.0%; spontaneous 77.4 ± 15.7%; p_anova = 2.10^-7^. Static square-wave: stimulus direction 8.9 ± 6.9%; opposite direction 4.0 ± 2.7%; spontaneous 87.1 ± 8.7%; p_anova = 8.10^-13^. Static missing-fundamental: stimulus direction 8.0 ± 7.5%; opposite direction 5.1 ± 1.7%; spontaneous 86.9 ± 8.3%; p_anova=8.10^-13^. Static 3rd harmonic stimulus: stimulus direction 5.4 ± 7.9%; opposite direction 12.6 ± 12.4%; spontaneous 82.0 ± 19.0%; p_anova = 1.10^-7^. Moving square-wave: stimulus direction 89.0 ± 8.7%; opposite direction 1.1 ± 1.8%; spontaneous 9.9 ± 7.3%; p_anova=5.10^-13^. Moving missing-fundamental: stimulus direction 1.4 ± 1.8%; opposite direction 84.2 ± 9.9%; spontaneous 14.4 ± 9.2%; p_anova = 2.10^-11^. Moving 3rd harmonic stimulus: stimulus direction 0.6 ± 1.5%; opposite direction 83.7 ± 15.0%; spontaneous 15.7 ± 14.3%; p_anova = 8.10^-9^. See also Supplementary Table 2 for more detailed statistical values.

The visual stimulation paradigm was composed of 4 min of a black screen to account for spontaneous activity baseline, followed by the 3 different stimuli (square-wave, missing-fundamental and 3rd harmonic signals, Figure 1A) were projected 20 times in each direction (left or right) in a random order. Each time the stimulus was projected for 8 s without movement (static) then it was moved either to the left or the right for 12 s, to avoid any interference of the potentially induced motion aftereffect by the previous moving stimulus (Pérez-Schuster et al., 2016).

To test whether the missing-fundamental signal depends on the orientation of the stimulus, we used the same missing-fundamental signal as before but moving in one of the 4 orthogonal directions (down, up, left or right). Each of these 4 directions of movements was presented to the larvae 10 times for 30 s, separated by 30 s of a black background and 30 s of a static missing-fundamental signal (horizontal or vertical according to the direction), and we presented also 10 times 30 s of a static square-wave stimulus.

### 2.4. Behavioral Assay

To monitor the visually induced eye rotations, we placed the larva in a drop of 2% low-melting point agarose in the center of a recording chamber. The agarose around the eyes was carefully removed to allow the eyes to rotate freely. The visual stimuli were projected on a screen (#216 White Diffusion, Rosco Cinegel) around the chamber using a pico-projecter (AAXA P4X). To record the eye movements, we illuminated the larva with a infrared LED (820 nm) and placed, above the chamber, an infrared video camera (DMK 22BUC03, The Imaging Source). To synchronize the video recordings with the visual stimuli we used an arduino board. Using the Bonsai program (Lopes et al., 2015), we first converted the original image into a binary one by thresholding the image. We then calculated the orientation of each eye by measuring the orientation of the ellipsoid corresponding to each eye against an arbitrary x axis. As this angle depended on the orientation of the larva, the average for each eye was then subtracted to obtain a zero baseline for all larvae. Using a semi-automatic custom-built program in Matlab (The MathWorks, Inc), we detected the saccades from the eye orientation traces and calculated the orientation of each saccade (Pérez-Schuster et al., 2016). The behavior was then sorted automatically into those three types: either spontaneous, pursuits to the right or pursuits to the left. If two consecutive saccades were alternating in their direction, the behavior was classified as spontaneous rotations. If two consecutive saccades were both going from the left to the right, the pursuits were from the right to the left thus the behavior was sorted as pursuits to the left. Inversely, if two consecutive saccades were from the right to the left, the behavior was classified as pursuits to the right.

### 2.5. Two-Photon Ca2+ Imaging

For the two-photon Ca2+ recordings we used the same approach as for the behavioral essays, however the eyes were not released from the agarose. The two-photon system consisted of a modified version of the MOM (Movable Objective Microscope) system (Sutter Instruments) with a 25x NA 1.05 Olympus objective and a Mai-Tai DeepSee Ti:sapphire laser (Spectra-Physics) tuned at 920 nm. The output power at the focal plane was less than 3 mW. The emission of the GCaMP signal passed through a FF705 dichroic filter, an AFF01-680 short-path filter (IR Blocker), and an FF01 520/70 band-pass filter (all from Semrock), and collected by a photomultiplier tube (H1070 GaAsP from Hamamatsu). The emission signal was pre-amplified with a SR-570 (Stanford Research Systems) and reconstituted and saved using *ScanImage 3.8* software (Pologruto et al., 2003) in Matlab (The MathWorks, Inc.). To synchronize the neuronal recordings with the visual stimuli we used an arduino board. We recorded from the optic tectum and the pretectum at an acquisition rate of 3.91 Hz, with 256x256 pixels resolution.

### 2.6. Data Analysis of Ca2+ Dynamics

#### 2.6.1. Registration

During the recording, a custom-made plugin for ScanImage allowed us to compensate online for any possible drift in the Z plane, by calculating every 100 frames the correlation of the plane being imaged with the first imaged plane and two other planes 2.2 μm dorsal and ventral to the initial plane. If the correlation was greater with another plane than the original one, the stage was moved up or down accordingly by 0.44 μm. If the imaged sequences displayed drifts in the ventro-dorsal direction despite this online curation, the experiment was discarded. The series of images during a given experiment were saved as TIFF stacks (10,900 frames). To compensate for possible slow drifts in the XY plane, we registered the stacks using the Image J plugin Template Matching, in combination with a custom-made algorithm (Matlab, The MathWorks, Inc.) to further smooth the registration.

#### 2.6.2. Movement Artifacts

Movement artifacts were detected according to large deviations in the cross-correlation between successive frames. All frames with large deviations (z-score smaller than -3) were then manually inspected. Due to the agarose elasticity, the imaging plane almost invariantly returned to its original position, after observing movement artifacts. If this was not the case, the complete experiment was discarded. For the subsequent data analysis, we did not include frames showing moving artifacts. In average, we detected 0.24% ± 0.08% of the total frames having moving artifacts.

#### 2.6.3. Segmentation

Regions of interest (ROIs) corresponding to the imaged neurons were semi-automatically detected based on morphology according to a watershed algorithm (Romano et al., 2017). Because the fluorescence of the H2B-GCaMP6f is located in the nuclei, the algorithm identified neurons by finding local fluorescence intensity peaks. This program produced putative ROIs layouts that were afterwards manually curated. We then computed the changes in calcium associated to the activity of each imaged neuron by averaging the fluorescence of all pixels within the ROIs, across time.

#### 2.6.4. Detection of Significant Calcium Events

The baseline of the time series of each neuron was computed as the 8th percentile in a 30 s-long running time window to obtain the slow fluctuations unrelated to the fast calcium transients associated with the neuronal activity (Romano et al., 2017). The relative change in fluorescence (ΔF/F) corresponded to the difference between the fluorescence at each point in time and the baseline fluorescence. A data sanity test discarded ROIs with fluorescence signals too low and/or presenting artifactual fluorescence traces, i.e., sudden variation of the baseline fluorescence (as in unhealthy or dying neurons, or healthy neurons that drifted in and out of focus). In average, we discarded 3.6 ± 9.1% of the originally segmented neurons.

In order to infer the calcium related fluorescence events associated with neuronal activity, we calculated the statistical significance of single-neuron calcium dynamics in an adaptive and unsupervised manner (Romano et al., 2015, 2017; Pérez-Schuster et al., 2016). We considered that any event in the fluorescence time series data belongs to either a neuronal activity process or to an underlying noisy baseline. In order to discriminate, with a desired degree of confidence, between these two sources, we built a data-driven model of the noise (Romano et al., 2017). Moreover, we took into account the biophysical constraints of the fluorescent calcium indicator (H2B-GCaMP6f fluorescence time constant 2.88 s Kawashima et al., 2016). Then, we applied a Bayesian odds ratio estimation framework. This method labels as significant with at least 95% confidence the fluorescence data points whose dynamics meet two conditions: i) it cannot be explained by the underlying fluorescence noise; ii) they are compatible with the H2B-GCaMP6f time constant. We obtained significantly and non-significantly active portions of the ΔF/F traces. A more detailed explanation of the calculation significance can be found in Romano et al. (2015).

#### 2.6.5. Determination of the Neurons Responsive to the Visual Stimuli

To find the neurons that were responsive to each type of presented visual stimulus, we measured the mean activity for each ROI:


mean activity during stimulus-mean activity during black

and from that the zscore:


$$zscore=\frac{mean\;activity-mean\;activity\;of\;all\;ROIs}{standard\;deviation\;of\;all\;ROI}$$

and discarded the ROIs that had a z-score inferior to –1. Then the ROIs were considered responsive if they showed at least 4 frames of significant activity (approximately 1 s) during the period of stimulation, in at least half of the repetitions of that stimulus.

#### 2.6.6. Spatial Location of the Responsive Neurons

We recorded the neuronal activity from several larvae and we used custom-made algorithms in Matlab (The MathWorks, Inc.) to register all the neurons from the different larvae on the same reference brain. We chose a reference pretectal and tectal plane from a specific larva and calculated for each other larva the affine transformation that was necessary for aligning the individual plane to the reference one, with the help of anatomical landmarks. For the analysis, the neurons of interest from every larva could then be projected on the same reference brain.

### 2.7. Statistical Analysis and Reproducibility

To quantify and statistically compare the 3 types of behavior (spontaneous rotations, pursuits in the stimulus direction, pursuits in the opposite direction), we ran a One-Way ANOVA. When the p_anova value was less than 0.05, we ran a multiple comparison analysis between the different behaviors. We obtained a 95% confidence interval for each of the comparison and the associated *p*-values.

To assess the differences in the percentage of tectal vs. pretectal neurons that display the different response types, we used the non-parametric Wilcoxon rank sum test. To avoid false positive results that could happen due to the multiple number of response types, we corrected the *p*-values with the False Discovery Rate method from Benjamini and Hochberg (1995).

All values are reported as means ± SD throughout.

## 3. Results

When confronted with moving visual stimuli, the majority of organisms follow the Fourier motion energy or luminance information, independently of other stimulus features such as edges or contrast. To investigate how Fourier components of moving visual stimuli are represented in the optic tectum and the pretectum (the main visual regions of the zebrafish brain), we presented to transgenic zebrafish larvae expressing pan-neuronally the genetically encoded calcium indicator GCaMP6f (Huc:H2B-GCaMP6f), vertical square-wave gratings and the corresponding missing-fundamental signal while monitoring neural circuits calcium dynamics by means of two-photon microscopy (see Materials and Methods).

To learn about the behavioral relevance of these two types of visual stimuli, we first monitored the eye movements along the horizontal plane (yaw) of the larva in the presence of square-waves or missing-fundamental stimuli. We classified the possible eye movements into three types: pursuits in the direction of the stimulation, pursuits in the opposite direction of the stimulus and spontaneous eye movements (see Materials and methods). The stimulus paradigm consisted of projecting a black background for 5 min, then the static patterns of square-wave, missing-fundamental or 3rd harmonic stimuli for 5 min, and 5 min of moving square-wave, missing-fundamental or 3rd harmonic signals (Figure 1A). In parallel, we recorded the induced eye rotations of the larvae using a video camera (Figure 1B and Supplementary Video 1, see section 2).

During the black background presentation, the majority of the behaviors consisted of spontaneous eye rotations. Similar results were observed during the static square-wave, the static missing-fundamental or the static 3rd harmonic stimulus. During the moving square-wave stimulus, the majority of behaviors consisted of pursuits in the direction of the stimulus. During the presentation of the moving missing-fundamental stimulus the majority of eye rotations consisted of pursuits in the opposite direction of the stimulus. The same was observed for the moving 3rd harmonic stimulus (Figure 1C and Supplementary Video 1, Supplementary Table 2).

Therefore, motion detection in the zebrafish larva seems to follow the direction of the Fourier energy and not other stimulus' features (edges, contrast), as was previously observed using the optomotor response (Orger et al., 2000).

### 3.1. Visual Responses in the Optic Tectum and the Pretectum

To study the neuronal responses induced by the square-wave and the missing-fundamental stimuli, we performed two-photon calcium imaging of a dorsal optical plane of the optic tectum (*n* = 13 larvae), and of an optical plane of the pretectum (*n* = 7 larvae), while presenting to the larva the different types of visual stimuli. In addition to the square-wave signal and the missing-fundamental signal, we also projected to the larva a square-wave with the spatial frequency of the 3rd harmonic of the original square-wave (see Figure 1A). The latter served as a control since the larva detects its movement in the same direction as the missing-fundamental stimulus, but all of its features move in the same direction.

**Figure 2:**
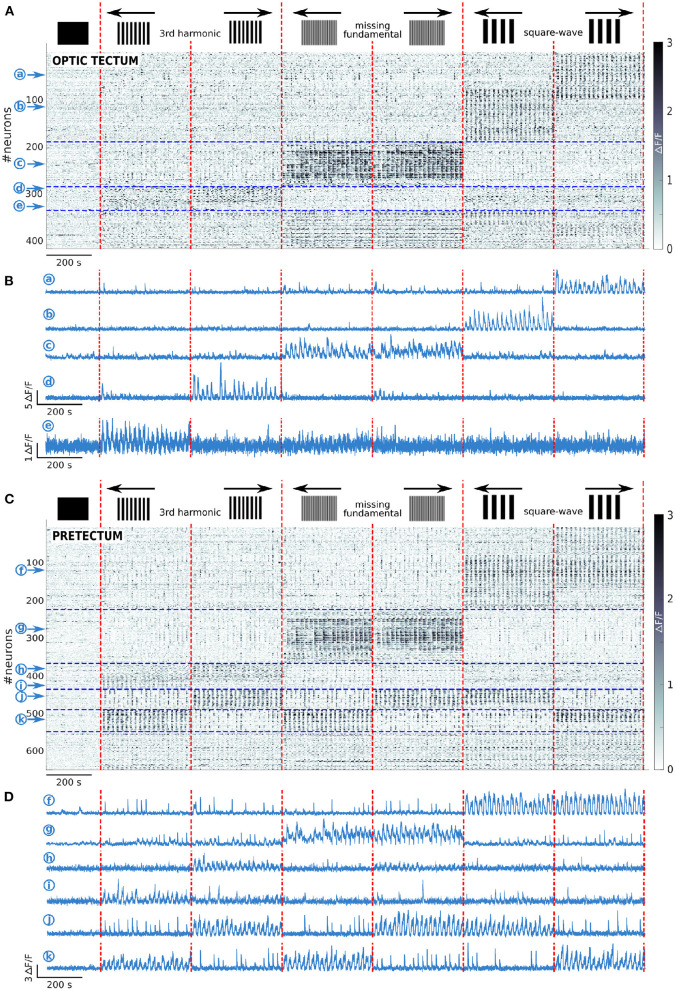
Tectal and pretectal neuronal representation of the visual stimuli. **(A)** Raster of activity of the neurons responding to at least one of the presented stimuli in the optic tectum (*n* = 13 larvae). The imaged frames are sorted on the x axis so that stimuli of the same type are grouped together (separated by vertical red dashed lines). The neurons are sorted on the y axis according to the type of response they display (separated by horizontal blue dashed lines). Note that the frequency in the Ca2+ signal observed during the presentation of the stimulus is due to the alternation between static and moving stimuli. **(B)** Examples of the neuronal responses of 5 neurons of the optic tectum induced by the square-wave stimulus to the right (a), or the left (b), the missing-fundamental stimulus in both directions (c), the 3rd harmonic stimulus to the right (d), or the left (e). **(C)** Same as **(A)** but for the pretectum (n = 7 larvae). **(D)** Examples of the stimulus-induced activity of 6 neurons in the pretectum that responded to the square-wave stimulus in both directions (f), to the missing-fundamental stimulus in both directions (g), to the 3rd harmonic stimulus to the right (h), or to the left (i), according to the behavioral output to the left (j, 3rd harmonic and missing-fundamental stimuli to the right and square-wave stimulus to the left), according to the behavioral output to the right (k, 3rd harmonic and missing-fundamental stimuli to the left and square-wave stimulus to the right).

To classify the recorded neurons into groups according to their response patterns to the different features of the presented visual stimuli, we calculated for each neuron its z-score and the number of frames that it was significantly active during each of the presented stimuli (see Materials and Methods). The neurons were considered as responding to a given stimulus: 1) if they had a z-score >-1, 2) if they were active for at least 1s during the presentation of the stimulus, 3) if they were active for at least half of the repetitions of the stimulus. We then clustered the neurons according to their type of response. Most neurons were selectively responsive to a subset of the 6 different moving visual stimuli (Figure 2).

In the optic tectum, across all larvae, we found 3 neuronal groups (Figures 2A,B): 1) Neurons specifically responding to the square-wave stimulus to the right (18.6% of the responding neurons) and to the left (21.4%), with a small fraction of neurons responding to both directions (5.5%); 2) Neurons specifically responding to the missing-fundamental stimulus in both directions (15.0%), with just a small fraction responding to the right (4.3%) or to the left (3.6%); and 3) Neurons specifically responding to the 3rd harmonic stimulus to the right (6.7%) or to the left (3.8%). Other types of neurons represented less than 2% of the responding neurons. In the latter group, we found neurons responding unspecifically to all stimuli, neurons responding to the missing fundamental and the square-wave stimuli, and others responding to the square-wave signals independently of their spatial frequency. In total, the responding neurons to the different types of presented stimuli in the optic tectum represent 4.8 ± 0.4% of all recorded neurons.

We then reclassified the neurons according to their responses to the static and moving part of the presented stimuli (see Materials and Methods). We found that neurons responding to the square-wave and 3rd harmonic stimuli, responded specifically to the moving part of the stimulus. In contrast, most of the neurons responding to the missing-fundamental stimulus in both directions responded to the static part of the stimulus (61.6%), or to both the static and moving parts (36.0%) (Supplementary Figures 3A,C). Only a very small percentage responded specifically to the moving part (2.4%). We thus suggest that for the missing-fundamental stimulus, the tectal neurons mainly responded to the specific contrast pattern of the stimulus rather than to its motion (the local contrast in the missing-fundamental signal is different from that of the square-wave and the 3rd harmonic stimuli). This response does not represent a motion illusion induced by the static missing-fundamental signal since this stimulus induced spontaneous eye rotations rather than pursuit movements (Figure 1C). Overall, directional neurons in the optic tectum respond to moving visual stimuli when the Fourier energy and other movement feature are coherent. This directional response also depends on the spatial frequency of the stimulus.

In the pretectum, we found 5 neuronal groups (Figures 2C,D): 1) Neurons specifically responding to the square-wave stimulus to the right (11.7% of the responding neurons), to the left (8.8%), or to both directions (13.4%); 2) Neurons specifically responding to the missing-fundamental stimulus in both directions (13.3%), to the right (3.7%) or to the left (5.3%); 3) Neurons specifically responding to the 3rd harmonic stimulus to the right (4.2%) or to the left (4.8%); 4) Neurons responding to the square-wave to the left, the missing-fundamental to the right and the 3rd harmonic stimuli to the right (5.4%, corresponding to the larva's behavioral output: pursuits eye movements to the left), or just to the missing-fundamental and 3rd harmonic stimuli to the right (2.9%, corresponding to the behavioral output induced by the 3rd harmonic frequency going to the left); and 5) Neurons responding to the square-wave to the right, the missing-fundamental to the left and the 3rd harmonic stimuli to the left (5.4%, behavioral output to the right), or just to the missing-fundamental and 3rd harmonic stimuli to the left (3.7%, corresponding to the behavioral output induced by the 3rd harmonic frequency going to the right). Other types of neurons represented less than 2% of the total number of responding neurons. Overall, the responding neurons to the different types of presented stimuli in the pretectum represent 10.1 ± 1.3% of all recorded neurons. The groups 4) and 5) are populations of neurons that we did not observe in the optic tectum and that represent the Fourier motion direction independently of the other motion features of the stimulus (second order features), and also independently of the spatial frequency of the signal, corresponding to the behavioral output (following the direction of motion).

In the pretectum, we found that the neuronal population responding to the missing-fundamental stimulus in both directions was mostly responding to both the static and the moving part of the stimulus (67.0%), with less neurons responding only to the static part (24.1%) or only to the moving part (8.9%) (Supplementary Figures 3B,C).

**Figure 3:**
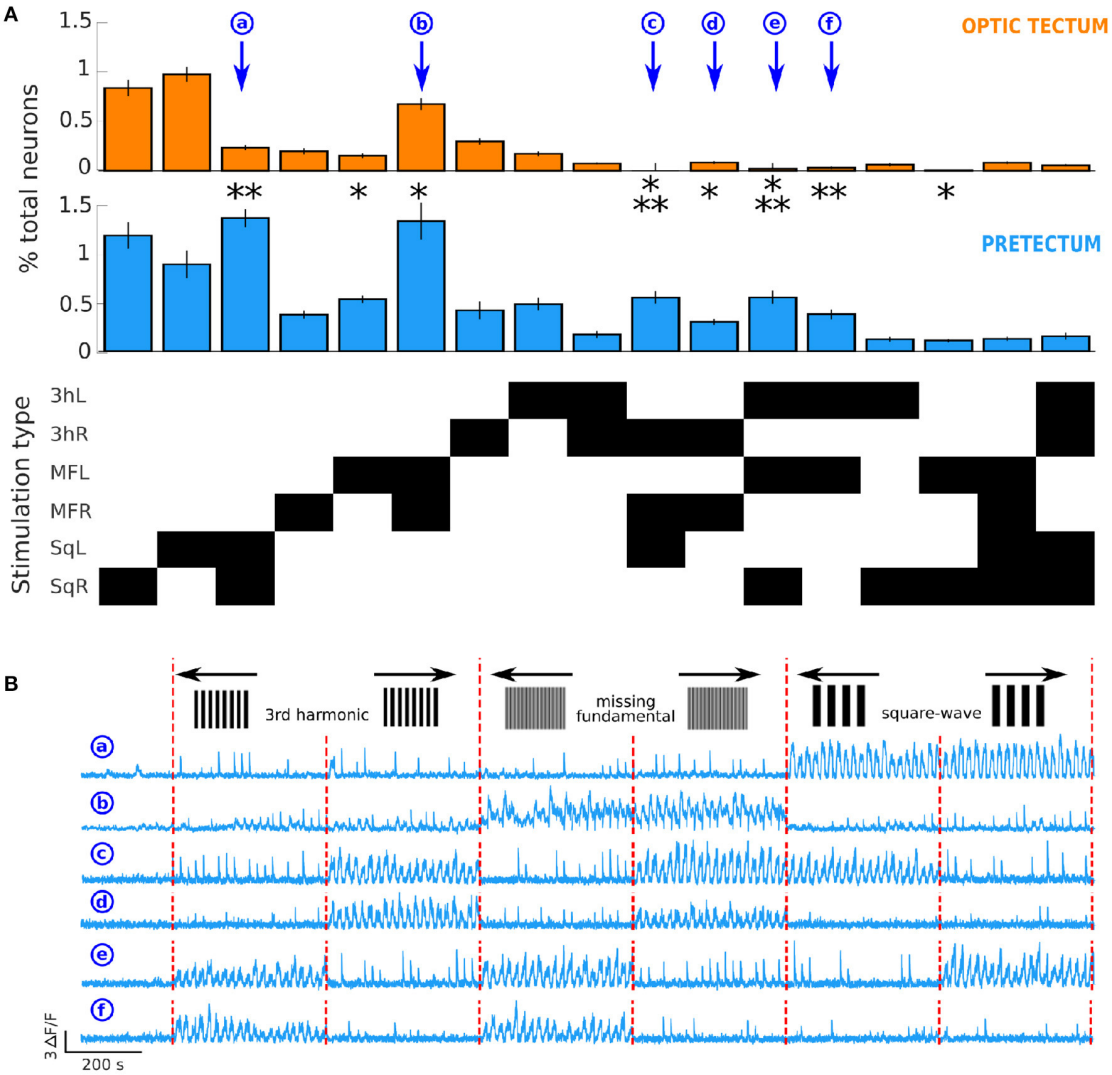
Differences in neuronal response types in the optic tectum and the pretectum. **(A)** Top: proportion of neurons responsive to the different types of presented stimuli in the optic tectum (*n* = 13) and Middle: in the pretectum (*n* = 7). Bottom: The response types that involved ≥ 0.1% of the total recorded neurons in the optic tectum or the pretectum. The stimulation types are represented on the y axis, the black rectangles indicate the type of stimulation represented in top and middle panels. 3hL: 3rd harmonic signal to the left; 3hR: 3rd harmonic signal to the right; MFL: missing-fundamental signal to the left; MFR: missing-fundamental signal to the right; SqL: square-wave signal to the left; SqR: square-wave signal to the right. Wilcoxon rank sum test corrected with the false discovery rate: *p ≤ 0.05, **p ≤ 0.01, ***p ≤ 0.001. Values as means±S.D.: Response to SqL and SqR (a): pretectum 1.34 ± 0.18% of all recorded pretectal neurons, optic tectum 0.24 ± 0.05% of all recorded tectal neurons, *p* = 0.002. Response to MFL: pretectum 0.52 ± 0.08%, optic tectum 0.16 ± 0.05%, *p* = 0.014. Response to MFL and MFR (b): pretectum 1.31 ± 0.99%, optic tectum 0.68 ± 0.43%, *p* = 0.046. Response to 3hR, MFR and SqL (c): pretectum 0.54 ± 0.13%, optic tectum 0 neurons, *p* = 5.10^-4^. Response to 3hR and MFR (d): pretectum 0.30 ± 0.06%, optic tectum 0.08 ± 0.03%, p = 0.018. Response to 3hL, MFL and SqR (e): pretectum 0.54 ± 0.13%, optic tectum 0.02 ± 0.02%, *p* = 7.10^-4^. Response to 3hL and MFL (f): pretectum 0.37 ± 0.10%, optic tectum 0.03 ± 0.02%, *p* = 0.006. Response to MFL and SqR: pretectum 0.11 ± 0.09%, optic tectum 0.01 ± 0.03%, *p* = 0.014. **(B)** Examples of the activity of 6 neurons of the pretectum that respond, respectively to the square-wave signal moving in both directions (a), to the missing-fundamental signal moving in both directions (b), according to the behavioral output to the left (c, 3rd harmonic and missing-fundamental signals to the right and square-wave signal to the left), to the 3rd harmonic and the missing-fundamental signals to the right (d), according to the behavioral output to the right (e, 3rd harmonic and missing-fundamental signals to the left and square-wave signal to the right), or to the 3rd harmonic and the missing-fundamental signals to the left (f). Vertical red dashed lines separate the different types of presented stimuli.

The 6 different visual moving stimuli that we presented to the larvae could induce 2^6^ different types of neuronal responses. To quantify the differences in the neuronal response types in both the optic tectum and the pretectum, we selected the response types for which we found at least 0.1% of the total recorded neurons in the pretectum or the optic tectum. This criteria revealed 17 different types of responses from the 64 possible ones (Figure 3A).

We found that the pretectum responded with a significantly larger portion of neurons than the optic tectum for 4 different classes of stimulus combinations. 1) Non-direction selective responses to the square-wave signal (neuron (a) in Figure 3B). 2) Non-direction selective response to the missing-fundamental stimulus (neuron (b) in Figure 3B), or direction selective responses to the left. The fact that we did not find a significant difference for the responses to the missing-fundamental stimulus going to the right might be due to the recordings of a non-uniform neuronal population across the entire circuit. 3) Neurons that responded to the Fourier motion energy in one or the other directions, regardless of the type of stimulus presented. For example, neuron (c) that responded to movement to the left or neuron (e) that responded to movement to the right (Figure 3B). 4) Neurons that responded specifically to the Fourier motion of the missing-fundamental and 3rd harmonic stimuli to the right (neuron (d) in Figure 3B), or to the left (neuron (f) in Figure 3B). These neurons are probably specific to a precise band of spatial frequencies including the 3rd harmonic frequency but not the frequency of the fundamental of the square-wave signal. We also found a significant difference for a small portion of neurons in the response to the missing-fundamental signal moving to the left and the square-wave stimulus moving to the right.

For the response to both directions, the neurons that respond are by definition not direction-selective. For these types of responses (square-wave, missing-fundamental or 3rd harmonic stimuli in both directions) the neurons are not responding to Fourier energy but to other components that we can only guess (edges, contrast, or motion in no specific direction). We argue that the pretectum detect Fourier energy through the direction-selective neurons (responses to either one or the other direction): the amount of response to the missing-fundamental signal to the left, missing-fundamental signal to the right, 3rd harmonic signal to the left or 3rd harmonic signal to the right are equivalent. Moreover, the responses to all signals that display the same Fourier motion direction (square-wave to the left, missing-fundamental to the right and 3rd harmonic to the right for the Fourier motion to the left; and square-wave to the right, missing-fundamental to the left and 3rd harmonic to the left for the Fourier motion to the right) are significantly larger in the pretectum than in the tectum.

Overall, we found that the pretectum show more non-direction selective responses to the square-wave and missing-fundamental stimuli than the optic tectum, and better responds to the Fourier energy motion of moving visual stimuli than the optic tectum, including the missing-fundamental signal.

### 3.2. Spatial Organization of the Visual Responses

**Figure 4:**
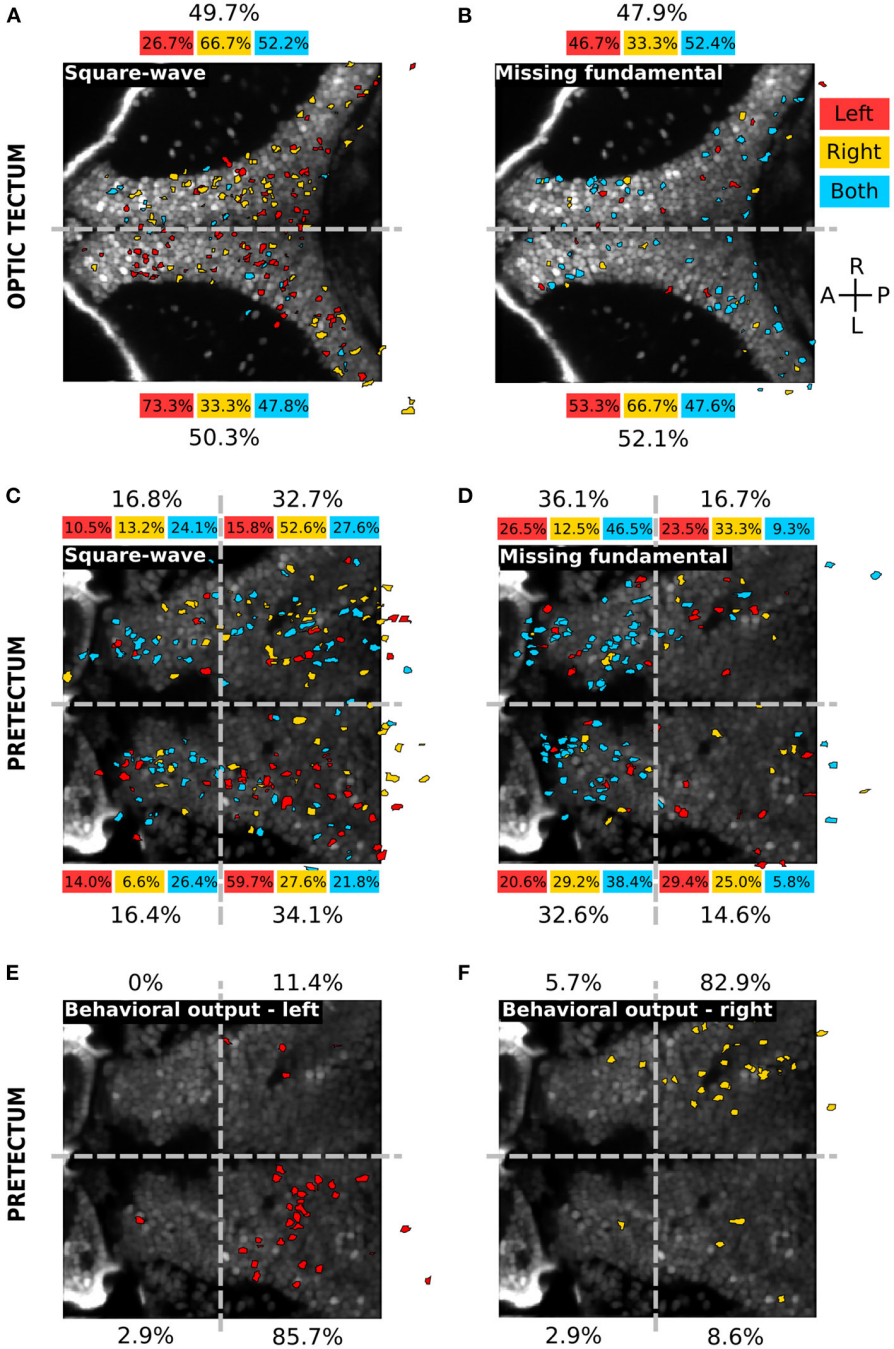
Spatial distribution of the different neuronal response types in the optic tectum and the pretectum. **(A)** Spatial distribution of the neurons responding to the square-wave signal to the left (red), to the right (yellow) or to both directions (cyan), in the optic tectum (*n* = 13). The percentages on top and bottom indicate the proportion of responsive neurons in the right and left hemispheres, for the responses to the left (red), to the right (yellow), to both directions (cyan) or in global for the square-wave stimulus (black). Gray dashed line: midline. **(B)** Same as **(A)** for the missing-fundamental signal. Note that the responses to both left and right directions of the missing-fundamental signal (cyan) are mostly induced by the static missing-fundamental stimulus (Supplementary Figure 3A). **(C)** Spatial distribution of the neurons responding to the square-wave signal to the left (red), to the right (yellow) or to both directions (cyan) in the pretectum (*n* = 7). The percentages next to each quadrant indicate the proportion of responsive neurons in each region, for the responses to the left (red), to the right (yellow), to both directions (cyan) or in global for the square-wave stimulus (black). Gray dashed horizontal line: midline. Gray dashed vertical line: separates between the anterior and posterior part of the pretectum. **(D)** Same as **(C)** for the missing-fundamental signal. Note that the responses to both left and right directions of the missing-fundamental signal (cyan) are mostly induced by the static missing-fundamental stimulus (Supplementary Figure 3B). **(E)** Same as **(C)** according to the behavioral output to the left (response to 3rd harmonic and missing-fundamental signals to the right and square-wave signal to the left). **(F)** Same as **(C)** according to the behavioral output to the right (response to 3rd harmonic and missing-fundamental signals to the left and square-wave signal to the right).

To assess the topographic distribution of the responsive neurons to the different types of visual stimuli, we registered the position of all neurons of each larva to a single reference plane (see Materials and Methods) (Figure 4). In the optic tectum, the responses to the square-wave (Figure 4A) and the missing-fundamental signals (Figure 4B) were not spatially organized. The responsive neurons were sparsely distributed with the same fraction of neurons in both hemispheres (49.7% right vs. 50.3% left hemisphere for the square-wave signal, 47.9% right vs. 52.1% left hemisphere for the missing-fundamental signal). However, we observed a light lateralization of the responses to the direction of the square-wave moving stimulus (66.7% right vs. 33.3% left hemisphere for responses to the right, and 26.7% right vs. 73.3% left for responses to the left) (Figure 4A). In the pretectum, the responses to the square-wave signal were also sparsely distributed (49.5% right vs. 50.5% left hemisphere), with more neurons in the caudal (66.8%) than the rostral part (33.2%). We also observed a lateralization of the responses to the direction of the square-wave moving stimuli (65.8% right vs. 34.2% left hemisphere for responses to the right, and 26.3% right vs. 73.7% left for responses to the left) (Figure 4C). In contrast, the responses to the missing-fundamental signal were principally found in the rostral part of the pretectum (68.7%) rather than in the caudal part (31.3%), without showing a lateralization of the responses to the direction of motion (45.8% right vs. 54.2% left hemisphere for responses to the right, and 50.0% right vs. 50.0% left for responses to the left) (Figure 4D). When we looked at the spatial organization of neurons responding according to the behavioral output (i.e., the type of eye rotation induced by the stimulus), we observed a clear topographic structure. Neurons responsive to visual stimuli that induced pursuit eye movements to the left were localized in the caudal part of the left hemisphere (85.7% of neurons, Figure 4E). Neurons responsive to visual stimuli inducing eye movements to the right were found in the caudal part of the right hemisphere (82.9% of neurons, Figure 4F). Therefore, the neuronal representation of the direction of the square-wave signal is spatially organized for the pretectum and the optic tectum. In contrast, the representation for the static missing-fundamental stimulus and the behavioral output were spatially organized in the pretectum but not in the optic tectum.

### 3.3. Visual Orientation Responses to the Missing Fundamental Stimulus

**Figure 5:**
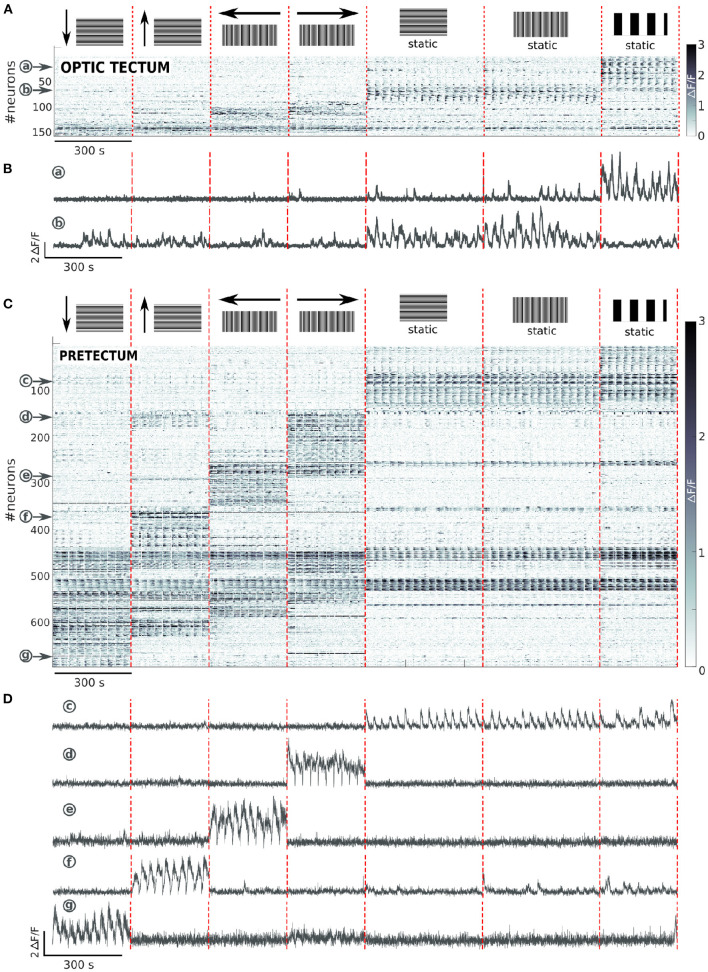
Tectal and pretectal neuronal representation of different directions of the missing-fundamental stimulus. **(A)** Raster of activity of the neurons responding to at least one of the 4 directions of the missing-fundamental signal, or to the static missing-fundamental or square-wave, in the optic tectum (*n* = 6 larvae). The imaged frames are sorted on the x axis so that stimuli of the same type are grouped together (separated by vertical red dashed lines). The neurons are sorted on the y axis according to the type of response they display (separated by horizontal blue dashed lines). **(B)** Examples of the activity of 2 neurons in the optic tectum that respond, respectively to the static square-wave signal (a), or to the static horizontal and vertical missing-fundamental (b). **(C)** Same as **(A)** for the pretectum (*n* = 6). **(D)** Examples of the activity of 5 neurons in the pretectum that respond, respectively to the static vertical and horizontal missing-fundamental signal and the static square-wave signal (c), or to the missing-fundamental signal going to the right (d), to the left (e), up (f) or down (g).

To investigate whether the observed neuronal responses to the missing-fundamental signal are dependent on the orientation of the stimulus, we presented to the larvae the missing-fundamental stimulus moving in 4 orthogonal directions: down, up (horizontal grating), left and right (vertical grating). We recorded from n = 6 larvae in the optic tectum and n=6 larvae in the pretectum. In the optic tectum, 4.5 ± 2.8% of the total recorded neurons were responsive to at least one of the directions or the static stimuli. We observed 2 types of neuronal responses (Figure 5A). 1) Neurons specifically responding to the static stimulus, either square-wave (34.4%, neuron a in Figure 5B) or horizontal (4.5%), vertical (4.5%) or both horizontal and vertical (7.0%, neuron b in Figure 5B) missing-fundamental signal. The latter neurons seemed to be responsive to the change of contrast and not the orientation. A few neurons also responded to any type of static stimuli (3.8%). 2) Neurons specifically responding to the different directions of orthogonal motion of the missing-fundamental stimulus: down (3.2%), up (2.5%), left (7.0%) or right (7.0%). Some neurons responded to both left and right directions (6.4%, orientation-selective neurons) but none were found to specifically respond both to up and down motion.

**Figure 6:**
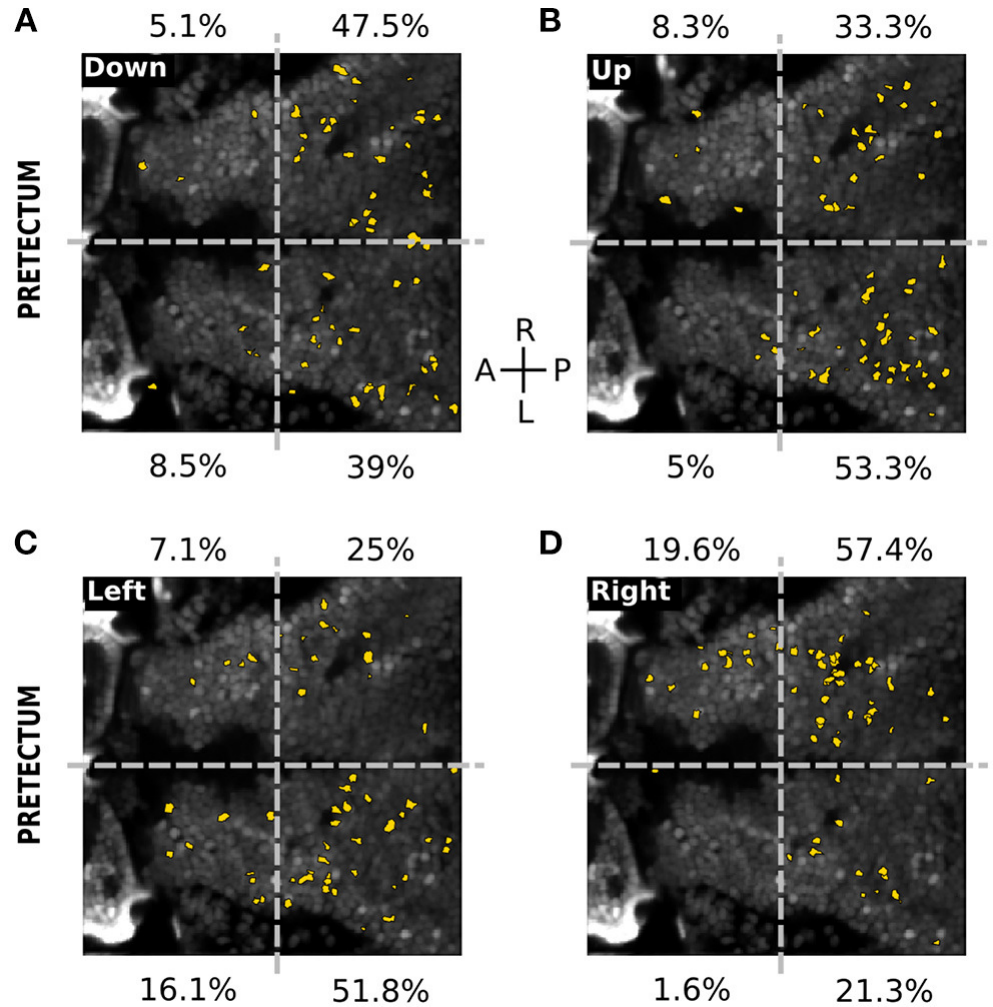
Spatial distribution of neurons responding to the directions of the missing-fundamental signal in the pretectum. **(A)** Spatial distribution of the neurons (yellow) responding to the horizontal missing-fundamental signal moving downwards in the pretectum. The percentages next to each quadrant indicate the proportion of responsive neurons in each region. Gray dashed horizontal line: midline. Gray dashed vertical line: separates between the anterior and posterior part of the pretectum. **(B)** Same as **(A)** for the horizontal missing-fundamental signal moving upwards. **(C)** Same as **(A)** for the vertical missing-fundamental signal moving leftwards. **(D)** Same as **(A)** for the vertical missing-fundamental signal rightwards.

In the pretectum 10.9 ± 2.6% of the total recorded neurons were responsive to at least one of the directions or the static stimuli. We observed 3 types of neuronal responses (Figure 5C). 1) Neurons specifically responding to the static stimulus, either square-wave (8.8%) or horizontal (1.0%), vertical (4.3%) or both horizontal and vertical (1.5%) missing-fundamental signal. Neurons also responded to all static stimuli (5.5%, neuron c in Figure 5D). 2) Neurons specifically responding to the different directions of orthogonal motion of the missing-fundamental stimulus: right (10.1%, neuron d in Figure 5D), left (9.3%, neuron e in Figure 5D), up (9.9%, neuron f in Figure 5D) or down (9.8%, neuron g in Figure 5D). Some neurons were also orientation-specific, responding either to up and down (6.0%) or left and right (3.6%) motion. Other neurons were responding to visual motion regardless of its direction or orientation (3.0%). 3) Neurons responding not specifically to all types of stimuli (static and moving) (4.8%). The pretectal neurons responding to the 4 directions of the missing-fundamental moving stimulus were principally distributed in the caudal region (86.5% caudal vs. 13.6% rostral for the down direction; 86.6% caudal vs. 13.3% rostral for the up direction; 76.8% caudal vs. 23.2% rostral for the left direction; 78.7% caudal vs. 21.2% rostral for the right direction) (Figure 6). The horizontal directions moving stimuli induced responses specific to the ipsilateral hemisphere (67.9% of the responses in the left hemisphere during motion to the left, and 77% of the responses in the right hemisphere during motion to the right), but the responses to vertical directions did not show this lateralization (52.6% of the responses in the right vs. 47.5% in the left hemisphere for down motion, and 41.6% in the right vs. 58.3% in the left hemisphere for up motion). It is possible that the vertical directions are represented in the dorso-ventral axis and therefore impossible to observe when recording from a single optical plane, or alternatively according to the cell identity in the caudal region (different neurons in the caudal area respond to the up or down stimuli).

Overall, the pretectum represents in a topographic manner the 4 orthogonal directions of the missing-fundamental signal, but the optic tectum does not.

## 4. Discussion

Several studies have demonstrated that the missing-fundamental stimulus induces motion perception in the direction of the Fourier energy in humans (Chen et al., 2005; Sheliga et al., 2005) and monkeys (Miura et al., 2006), and correlate with the behavioral output of the OKR in mice (Sugita et al., 2012) and the optomotor response (OMR) in zebrafish (Orger et al., 2000). Here, we showed that in zebrafish larvae, the presentation of a missing-fundamental moving visual stimulus induces OKR in the opposite direction of that induced by square-wave moving stimuli. The missing-fundamental stimulus has all motion features in the direction of the square-wave signal except for the Fourier energy which moves in the opposite direction. Thus, we suggests that OKR in zebrafish larvae principally follows the Fourier energy of the moving visual stimulus. We then investigated the neuronal representation of these two moving visual patterns (square wave vs. missing fundamental), in the two main visual centers of the larva: the optic tectum and the pretectum. We found that the activity in the pretectum mainly represents the eyes behavioral output regardless of the type of stimulus presented (square-wave, missing-fundamental or 3rd harmonic). Therefore, the pretectum represents the Fourier energy of the moving visual stimulus (in the missing-fundamental stimulus, the Fourier energy is the only feature that correlates with the direction of the induced eye pursuits). In contrast, the optic tectum responds to the missing-fundamental stimulus, but rather to its contrast pattern than to its motion direction (the tectum responds to the static missing-fundamental stimulus but not to its movement). Our results are coherent with the fact that the pretectum has been shown to be necessary and sufficient for the OKR (Kubo et al., 2014) and that ablation of the optic tectum only minimally affects the kinematics of OKR (Roeser and Baier, 2003). Moreover, the pretectum is one of the main regions involved in the detection of optic flow (Matsuda et al., 2021), responding to large-field motion stimuli (Kubo et al., 2014).

In the optic tectum, the spatial structure of the visual responses to the different types of visual stimuli showed no topographic organization. This is in line with the finding that direction-selective neurons in the optic tectum are not topographically organized (Romano et al., 2015). In contrast, the pretectum showed responses to the direction of motion in the caudal part of the pretectum more than the rostral part (Figures 4, 6). In line with our results, a rostrally located region of the pretectum has been shown to be involved in prey capture (Semmelhack et al., 2014), thus implying that the optic-flow responsive part of the pretectum is located in its caudal part. In this study, we found that the rostral part of the pretectum is responsive mainly to the static missing-fundamental stimulus (Figure 4), suggesting that this part is reacting to the contrast pattern rather than the Fourier energy of the stimulus. The caudal region of the pretectum responded differently according to the direction of motion: each caudal hemisphere responded to Fourier energy toward one direction (left caudal: motion to the left—right eye moves in the temporal to nasal direction, and right caudal: motion to the right—left eye moves in the temporal to nasal direction). These observations are in agreement with the results obtained by Chen et al. (2016) showing that eye pursuits were longer in the temporal-to-nasal direction than the nasal-to-temporal direction, and Wang et al. (2019) that showed that pretectum's cells respond more to temporal-to-nasal than nasal-to-temporal monocular moving stimuli, together with the fact that the retina in zebrafish projects almost exclusively to the contralateral hemisphere of the brain (Burrill and Easter, 1994).

Finally, the optic tectum responded specifically to the static square-wave and missing-fundamental stimuli. In contrast, in the pretectum, the neurons responding to the static square-wave stimuli also responded to the static missing-fundamental.

Overall, we suggest that the optic tectum represents multiple features of visual motion (the Fourier motion but also the contrast, texture, edges, etc.). In case that Fourier energy does not coherently move in the direction of other features, the optic tectum cannot represent the direction of motion. In contrast, the pretectum's activity, mainly in its caudal part, represents the optic flow principally based on the Fourier energy information. A previous study showed that zebrafish larvae follow, using the OMR, second-order motion in the absence of Fourier content (Orger et al., 2000), thus it is possible that when confronted to only second-order motion, the optic tectum rather than the pretectum controls OKR and OMR. In conclusion, we suggest that the optic tectum plays a role in the extraction of the different features of static (contrast patterns and spatial frequency) and moving stimuli (Fourier and second-order features), while the pretectum mainly responds to the Fourier energy of a moving visual stimulus to generate OKR and OMR.

## Data Availability Statement

The toolbox used for the analysis of calcium dynamics is detailed in Romano et al. (2017) and is available in Github at this address https://github.com/zebrain-lab/Toolbox-Romano-et-al. The datasets as well as data analysis programs generated specifically for this study are available on request to the corresponding author.

## Ethics Statement

The animal study was reviewed and approved by Comité d'éthique en expérimentation animale n°005. Reference number APAFIS#27495-2020100614519712 v14.

## Author Contributions

AD designed the experiment, recorded and analyzed data, and wrote the manuscript. MP wrote analysis programs and discussed data analysis. GS designed the experiment, advised data analysis, and wrote the manuscript. All authors contributed to the article and approved the submitted version.

## Funding

This research was supported by the Labex Memolife, and the ENS Paris-Saclay Ph.D. fellowships to AD, the Fondation pour la Recherche Medicale and the ENS Lyon Ph.D. fellowship to MP, and the ERC CoG 726280 to GS. The funders had no role in the study design, data collection and analysis, decision to publish, or preparation of the manuscript.

## Conflict of Interest

The authors declare that the research was conducted in the absence of any commercial or financial relationships that could be construed as a potential conflict of interest.

## Publisher's Note

All claims expressed in this article are solely those of the authors and do not necessarily represent those of their affiliated organizations, or those of the publisher, the editors and the reviewers. Any product that may be evaluated in this article, or claim that may be made by its manufacturer, is not guaranteed or endorsed by the publisher.
