# Supplementary material for: Fourier Motion Processing in the Optic Tectum and Pretectum of the Zebrafish Larva
Source: Front Neural Circuits. 2022 Jan 7;15:814128. doi: 10.3389/fncir.2021.814128 (PMC8777272; doi:10.3389/fncir.2021.814128)
Supplement: Supplementary file 1 [file Data_Sheet_1.PDF]

Supplementary Material

1 SUPPLEMENTARY DATA

2 SUPPLEMENTARY TABLES AND FIGURES

2.1 Figures

Figure S1. (Video related to Figure 1) Behavioral eye output during the presentation of the static square-wave (left), the moving square-wave (middle) and the moving missing-fundamental (right) stimuli. The stimulus motion is rightwards. **(Top)** Visual appearance of the stimuli. **(Bottom)** Video of a 7dpf larva showing eye rotations induced by the visual stimuli. The larva performs spontaneous rotations during the static square-wave stimulus, pursuits to the right during the square-wave stimulus moving rightwards, and pursuits leftwards during the missing-fundamental stimulus moving to the right. The video is speeded up 2x with respect to the original recording.

**Stimulus**	black	square static	missing fundamental static	3rd harmonic static	square moving	missing fundamental moving	3rd harmonic moving
**p_anova**	2.10^{-7}	8.10^{-13}	8.10^{-13}	1.10^{-7}	5.10^{-13}	2.10^{-11}	8.10^{-9}
**F value**	51.23	299.28	301.34	55.86	320.67	190.99	82.11
**95%CI opposite vs samedir**	-29.8 : 9.7	-14.8 : 5.0	-12.7 : 7.0	-13.6 : 28.0	-97.8 : -77.9	71.0 : 94.6	65.2 : 101.1
**p_value**	0.41	0.42	0.73	0.65	1.10^{-9}	1.10^{-9}	1.10^{-8}
**95%CI samedir vs spontaneous**	-80.8 : -41.3	-88.0 : -68.3	-88.8 : -68.3	-97.4 : -55.8	69.1 : 89.0	-24.8 : -1.1	-33.0 : 2.9
**p_value**	2.10^{-6}	1.10^{-9}	1.10^{-9}	3.10^{-7}	1.10^{-9}	0.03	0.11
**95%CI opposite vs spontaneous**	-90.8 : -51.4	-93.0 : -73.2	-91.7 : -72.0	-90.3 : -48.7	-18.7 : 1.1	58.0 : 81.7	50.1 : 86.0
**p_value**	3.10^{-7}	1.10^{-9}	1.10^{-9}	9.10^{-7}	0.09	2.10^{-9}	2.10^{-7}

Figure S2. (Table related to Figure 1) Statistical values for the ANOVA tests used to compare the different eye behaviors (pursuits in the same direction than the stimulus, pursuits in the opposite direction,spontaneous rotations) induced by each of the presented stimuli. Row from top to bottom: Stimulus type; p_value of the ANOVA test; F value of the ANOVA test; 95% confidence interval for the comparison between the pursuits in the stimulus direction and the opposite direction, and the p_value associated; 95% confidence interval for the comparison between the pursuits in the stimulus direction and the spontaneous rotations, and the p_value associated; 95% confidence interval for the comparison between the pursuits in the opposite direction and the spontaneous rotations, and the p_value associated. Significant p_values (inferior to 0.05) are displayed in dark orange.

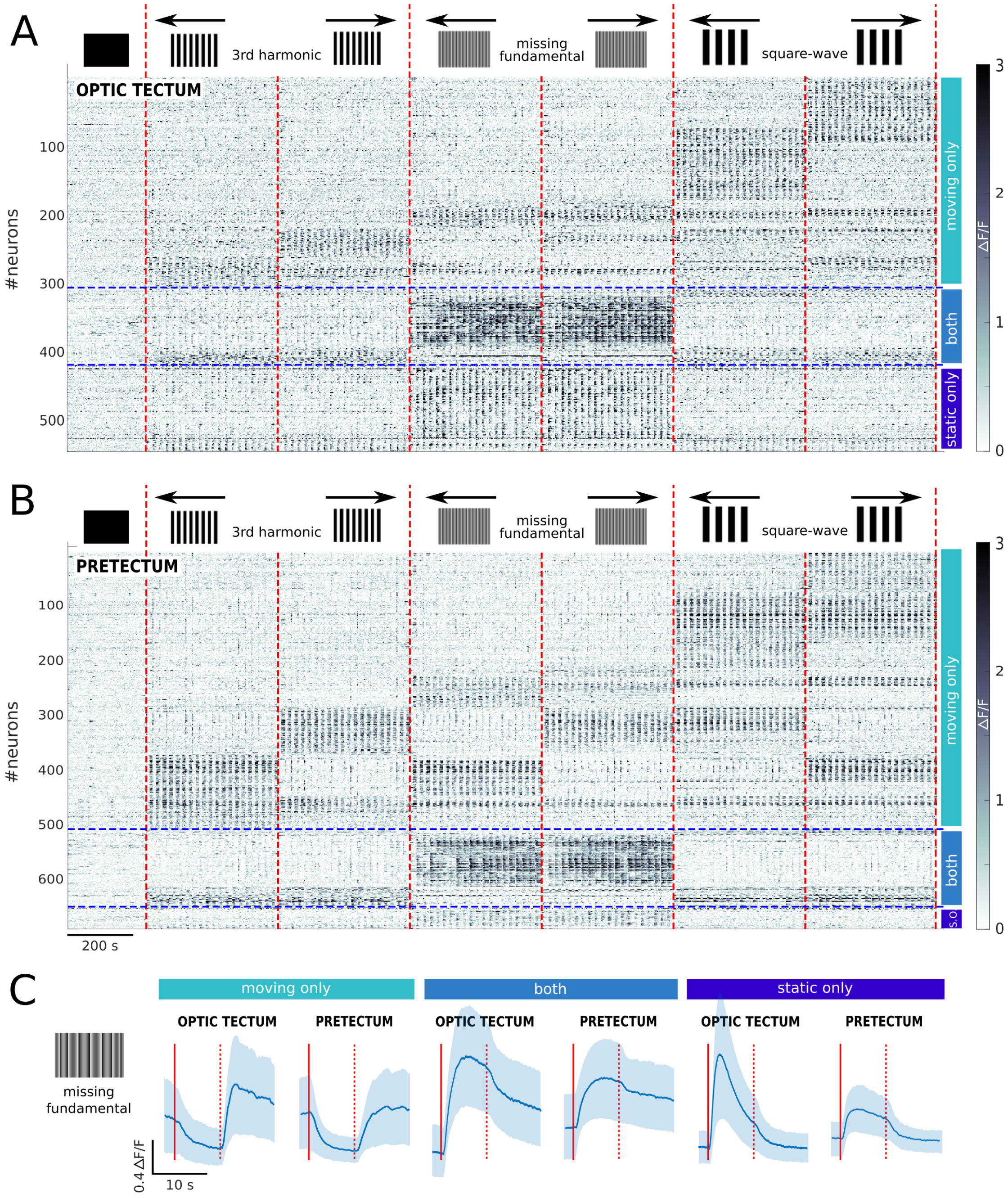

Figure S3. (related to Figure 2) Tectal and pretectal neuronal activity induced by the presentation of the different types of visual stimuli. **(A)** Raster of activity of the neurons responding to at least one of the presented stimuli in the optic tectum (n=13 larvae). The imaged frames are sorted on the x axis so that stimuli of the same type are grouped together (separated by vertical red dashed lines). The neurons are sorted on the y axis according to the type of response they display. The neurons are also separated in three categories (separated by horizontal blue dashed lines): 1) show activity only during the moving stimulus (cyan), 2) show activity only during the static part of the stimulus (purple), or 3) show activity during the static and the moving part (blue). Note that the frequency in the Ca2+ signal observed during the presentation of the stimulus is due to the alternation between static and moving stimuli. **(B)** Same as **A** but for the pretectum (n=7 larvae). S.O. in the purple box stands for static only. **(C)** Average of the activity of tectal and pretectal neurons responding only to the moving part of the missing-fundamental stimulus, to the moving and static part or only to the static part. Vertical red solid line: onset of the static stimulus. Vertical red dashed line: onset of stimulus motion. Light blue: standard deviation.
